# Melt-Mixed 3D Hierarchical Graphene/Polypropylene Nanocomposites with Low Electrical Percolation Threshold

**DOI:** 10.3390/nano9121766

**Published:** 2019-12-11

**Authors:** Thomas Gkourmpis, Karolina Gaska, Davide Tranchida, Antonis Gitsas, Christian Müller, Aleksandar Matic, Roland Kádár

**Affiliations:** 1 Innovation & Technology, Borealis AB, SE-444 86 Stenungsund, Sweden; 2 Department of Industrial and Materials Science, Engineering Materials, Chalmers University of Technology, SE-412 96 Gothenburg, Sweden or roland.kadar@chalmers.se (R.K.); 3 Innovation & Technology, Borealis Polyolefine GmbH, St.-Peter-Straße 25, 4021 Linz, Austria; davide.tranchida@borealisgroup.com (D.T.); antonis.gitsas@borealisgroup.com (A.G.); 4 Department of Chemistry and Chemical Engineering, Chalmers University of Technology, SE-412 96 Gothenburg, Sweden; christian.muller@chalmers.se; 5 Department of Physics, Chalmers University of Technology, SE-412 96 Gothenburg, Sweden; matic@chalmers.se

**Keywords:** graphene, polypropylene, electrical percolation, electrical conductivity, nanocomposites, melt mixing

## Abstract

Graphene-based materials are a family of carbonaceous structures that can be produced using a variety of processes either from graphite or other precursors. These materials are typically a few layered sheets of graphene in the form of platelets and maintain some of the properties of pristine graphene (such as two-dimensional platelet shape, aspect ratio, and graphitic bonding). In this work we present melt mixed graphene-based polypropylene systems with significantly reduced percolation threshold. Traditionally melt-mixed systems suffer from poor dispersion that leads to high electrical percolation values. In contrast in our work, graphene was added into an isotactic polypropylene matrix, achieving an electrical percolation threshold of ~1 wt.%. This indicates that the filler dispersion process has been highly efficient, something that leads to the suppression of the β phase that have a strong influence on the crystallization behavior and subsequent thermal and mechanical performance. The electrical percolation values obtained are comparable with reported solution mixed systems, despite the use of simple melt mixing protocols and the lack of any pre or post-treatment of the final compositions. The latter is of particular importance as the preparation method used in this work is industrially relevant and is readily scalable.

## 1. Introduction

Graphene-based polymer nanocomposites attracted significant scientific and commercial interest in recent years [1,2,3,4,5,6]. The main reason for this interest lies in the outstanding performance that high aspect ratio fillers can induce into a polymer matrix [7,8,9,10]. This performance increase in comparison with traditional filled systems (e.g., carbon black) allows for a wide range of potential industrial applications [11,12], like gas-barriers [13], supercapacitors [14], or solar cells [15].

The introduction of a filler in a polymer matrix can lead to an increase of mechanical properties (e.g., Young’s modulus) and in the case of carbonaceous fillers an increase of electrical conductivity. Both these properties are tightly linked to the type of filler and polymer combination. Depending on the targeted application the filler amount might be significant (e.g., for application that require high electrical conductivity the amounts of carbon black used can be in the range of 15–40 wt.%), something that has been seen to lead to an increase of the overall viscosity with potential processing and performance difficulties [16]. The way the filler is incorporated into the polymer matrix is of paramount importance as it affects the overall dispersion and the subsequent properties of interest. Dispersion is further inhibited by the tendency of the filler particles to cluster in larger agglomerates, something that is mainly driven by the existence of attractive interactions between the filler particles [17]. These effects are augmented in the case of high aspect ratio fillers like graphene by the extensive size of the primary particle. The agglomeration that takes place during mixing can be understood via filler–filler, filler–polymer, and polymer–polymer interactions, where the polymer mobility plays an important role on the facilitation of local and long range arrangements of the filler particles (of all sizes) in the polymer matrix [18].

The most economically advantageous method of dispersing a filler in a matrix, especially in a large scale industrial environment, is by melt mixing. Such methods have been successfully used for traditional carbonaceous fillers like carbon black [19], but due to the inherent agglomeration and extensive clustering of the primary particles the resulting systems tend to exhibit a high electrical percolation threshold [20,21,22] and increased brittleness [23,24,25]. Similarly, for high aspect ratio fillers like graphene the attractive interactions between the filler particles and the resulting level of clustering and agglomeration render melt mixing a real challenge [3]. A number of attempts of melt mixing of thermally reduced graphene in glassy [1,26] thermoplastic [27,28] and elastomeric [29] polymers has been reported, but the majority of the resulting compositions yield systems of significantly worse graphene dispersion in comparison with solvent-based mixing methods [2].

Here we must note that although graphene is commonly seen as a pristine monolayer of hexagonal carbon atoms, in reality, there is a whole class of materials that can be considered graphene-like. The initial distinction comes from the different preparation methods that can be classified as bottom-up and top-down. Bottom-up graphene can be produced via a variety of methods that include chemical vapor deposition [30], epitaxial growth [31], chemical conversion [32], and reduction of CO [33]. Top-down graphene summarizes the different processes that originate from separation and subsequent exfoliation of graphite and its derivatives. These methods offer significant financial advantages for large scale applications since graphite is a material of relative abundance and reasonably low price [2]. The most promising graphene derivatives originating from this type of production are expanded graphite (EG) and graphene oxide (GO). Thin forms of EG (~10 nm) known as graphene nanoplatelets (GNP) are already commercial and can be produced by different types of graphite intercalation [27,34]. In these systems the flexural rigidity and dimensions of the platelets is retained during preparation, thus allowing some element of high aspect ratio properties to be present even without complete exfoliation [27,35]. Probably the most promising of alternatives is based on exfoliation and reduction of GO, a process that can be performed using a number of alternative variations of the Staudenmaier [36] or Hummers [37] methods, where graphite is oxidized in the presence of strong oxidants followed by a sequence of reduction steps, mainly to restore its electrical conductivity.

Polypropylene (PP) is one of the most commonly-used polyolefins with a myriad of application uses ranging from everyday items like trays and bottles to high performance compounds suitable for medical, automotive, energy transport and storage, and pipe products [38]. Due to its commercial importance a number of studies have been undertaken on PP/graphene nanocomposites focusing mainly on electrical [27,39,40,41], thermal [42], and mechanical [43,44,45] performance. Electrical conductivity and especially the electrical percolation threshold is directly linked to the level of dispersion of the filler in the matrix [46]. As discussed previously, melt mixing can lead to significant agglomeration and inefficient dispersion. That can be seen by relatively high values of the electrical percolation threshold^3^. In previous studies on PP the electrical percolation threshold for melt mixed systems using a variety of mixing protocols and pre/post mixing procedures has been reported in the range 6–15 wt.% [27,39,40,41]. These values are significantly higher than theoretical predictions [47] and values obtained for near perfect dispersion of graphene in a variety of matrices using solvents and sonication protocols [2]. This indicates that the nominal aspect ratio of the filler is being reduced by the mixing procedures leading to significant agglomeration that increases the electrical percolation threshold. For most fillers in their bulk form including different variants of graphene, a significant level of clustering and agglomeration exists, and this has the potential to be still present after mixing. For any such system to be commercially viable, the initial agglomeration level needs to be significantly reduced, thus allowing for a reasonably good dispersion using melt mixing protocols. The use of simple melt mixing protocols is of paramount importance if one considers large-scale commercial applications. This is due to the need for efficient and relatively streamlined procedures in an environment where safety, throughput and material property consistency needs to be evaluated and kept as constant as possible at all times, without forgetting that the overall volumes associated with the process can range from a few kilograms to a few hundred tones.

In this work we demonstrate for the first time an industrially relevant system of polypropylene with a novel hierarchical graphene nanostructure, which is capable to achieve superior levels of dispersion as seen through the observed electrical percolation threshold. Furthermore, the mixing protocol used was kept intentionally simple as to explore the potential industrial scalability of the process. By utilizing these simple mixing protocols, we have managed to achieve dispersion levels that are on par with solution techniques, something that becomes obvious from the location of the electrical percolation threshold at the region of ~1 wt.%. The combination of superior dispersion and the design of the filler (highly de-agglomerated) allows us for the first time to get partial access to the primary graphene particles in a melt mixed system and study the effect it has on its electrical, thermal and mechanical properties. A comprehensive overview of the effect of the filler on the overall morphology, thermal, rheological, and mechanical properties is also presented and discussed.

## 2. Materials and Methods

**Figure 1:**
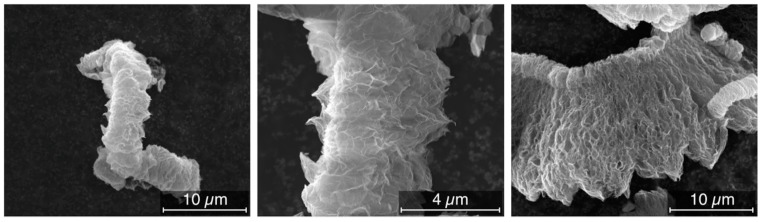
De-agglomerated hierarchical thermally reduced graphene oxide as received.

De-agglomerated hierarchical thermally reduced graphene oxide was supplied by Cabot Corporation. An overview of the available preparation methods for the different types of graphene can be found elsewhere [37]. In Figure 1 SEM pictures of the material in bulk form can be seen. From these pictures, we can see that despite the various production steps the material still contains a significant level of de-agglomeration.

A highly isotactic (>90%) polypropylene (M_w_ = 300 kg/mol, M_w_/M_n_ = 8) provided by Borealis AG was used.

### 2.1. Sample Preparation

All compositions were prepared using a Brabender mixer Type W50 driven by a Brabender Plasticorder. Initially the polypropylene was added and allowed to melt at 210 °C at 20 rpm for 15 min, followed by addition of graphene. The combined composite was then mixed at 210 °C at 50 rpm for another 15 min. Following compounding the composites were cut into small pellet-like pieces for easier use.

### 2.2. Electrical Measurements

Electrical conductivity measurements ware performed using a Novocontrol Alpha spectrometer in a frequency range of 10^−2^ to 10^7^ Hz, at atmospheric pressure and under nitrogen atmosphere. The sample cell consisted of two silver-coated electrodes 40 mm in diameter and the sample with a thickness of 0.1 mm. Each measurement was carried out six times, and average values were recorded. The complex conductivity σ* = σ′ + iσ″, the real part of which is used for the analysis herein, can be deducted from the complex dielectric permittivity ε* as σ* = iωε*_0_*ε*, where *ε*_0_ is the permittivity of free space. For compositions above the electrical percolation threshold, the conductivity value recorded was the one obtained from the frequency independent part of the curve, whereas for compositions below the electrical percolation the value recorded was the lowest conductivity value we were able to obtain as no conductivity plateau was reached within the experimentally available frequency window.

### 2.3. Thermal Analysis

DSC measurements were carried out under nitrogen at a rate of 10 °C/min in a temperature range of −50 and 210 °C, using a Mettler Toledo DSC2 equipped with a HSS7 sensor and a TC-125MT intercooler. The average weight of the samples was 3–4 mg.

Thermal Gravimetric Analysis, TGA, measurements were carried out using a Mettler Toledo TGA/DSC 3+ under nitrogen atmosphere with a heating rate 20 °C/min in a temperature range 30 °C −900 °C and kept at 900 °C for 10 min in air atmosphere.

### 2.4. Thermal Conductivity

Thermal conductivity was investigated using a Hot Disk Thermal Constants Analyser 2500 S following the ISO Standard 22007-2. All the measurements were carried out at room temperature and repeated 5 times for each sample and an average value was taken.

### 2.5. Rheology

Linear and nonlinear shear rheological tests were performed using an Anton Paar MCR702 TwinDrive (Graz, Austria) rotational rheometer in twin drive mode (separate motor-transducer) equipped with a convection oven (CTD450TD).

### 2.6. Melt Rheology

**Figure 2:**
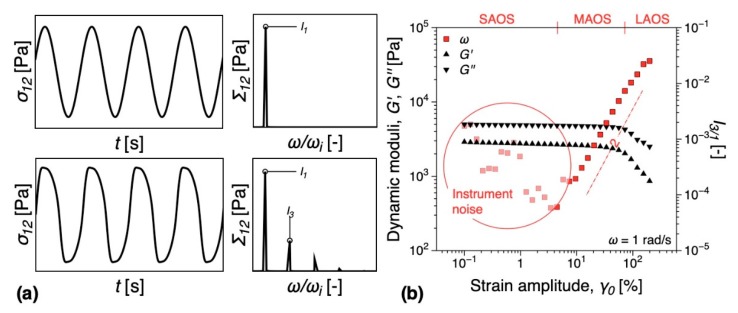
(**a**) Linear and nonlinear shear stress responses and their corresponding Fourier spectra. (**b**) Strain sweep test comparing the dynamic moduli, *G’*, *G’’*, and relative third relative higher harmonic, *I_3/1_*, for the iPP matrix. Notations: SAOS—small amplitude oscillatory shear; MAOS—medium amplitude oscillatory shear; LAOS—large amplitude oscillatory shear.

A 25 mm parallel plate geometry was used, with the gap set at 1 mm. The tests were performed at a measuring temperature of 200 °C. The nonlinear rheological analysis was performed using Fourier transform (FT) rheology, as a more sensitive method to detect rheological percolation [48,49,50,51]. Given a sinusoidal strain input, in contrast to linear viscoelastic oscillatory measurements, the shear stress response is non-sinusoidal and therefore higher harmonics are recording in the corresponding Fourier spectrum, Figure 2a. These higher harmonics give access to material nonlinear parameters, mainly through the use of the third relative higher harmonic, *I_3/1_*, as it contains the dominant nonlinear contribution to the shear stress signal [48]. The variation of *I_3/1_* during a strain sweep test on the iPP matrix investigated is presented in Figure 2b. At small strain amplitudes, the measured signal corresponds to instrumentation noise and indicates the sensitivity limits of the torque sensor. This region is called small amplitude oscillatory shear (SAOS). At a critical shear strain amplitude, the nonlinearities become detectable with *I_3/1_ ∝ γ^2^*, region called medium amplitude oscillatory shear (MAOS) or intrinsic nonlinearity. The large amplitude oscillatory shear regime (LAOS) is reached when the quadratic scaling with the strain amplitude is lost.

### 2.7. Dynamic Mechanical Analysis (DMTA)

A rectangular fixture (SRF12) was used with pressed samples of 50 mm in length, 10 mm in width and thickness varying between 0.8–1.5 mm. A heating rate of 2 °C/min was applied between −40 °C to 200 °C, the measurements were performed at 1 Hz.

### 2.8. Morphology

Scanning Electron Microscopy, SEM, was performed with a FEI Quanta 200. Fresh surfaces were prepared with a RMC ultracryomicrotome, and they were subsequently etched for one hour using a solution of 1 wt.% potassium permanganate in 86% ortho-phosphoric acid. The process was terminated by rinsing the samples with deionized water, followed by hydrogen peroxide and finally isopropanol. The etching has been performed in order to show clearly the iPP’s microstructure as well as the filler dispersion. Afterwards, approximately 5 nm thick Pd-Au layer was deposited onto the observed surfaces.

Electric Force Microscopy, EFM, was performed with an Asylum Research MFP-3D Atomic Force Microscope. Conductive tips, ASYELEC-01, were used. The samples were first scanned in AFM mode with free oscillation amplitude of ca. 1V and set point ratio of 0.7. The EFM scans were performed in nap mode. The tip tracked the sample surface, and was afterwards scanned again following the same profile, yet raised of 50 nm. In this second pass a voltage of 3 V was applied to the tip, and the tip scanned with very low amplitude. Electrical interaction between the sample and the DC voltage applied to the tip causes a force gradient which perturbs the oscillation frequency and as a result the phase shift.

## 3. Results and Discussion

### 3.1. Electrical Conductivity

**Figure 3:**
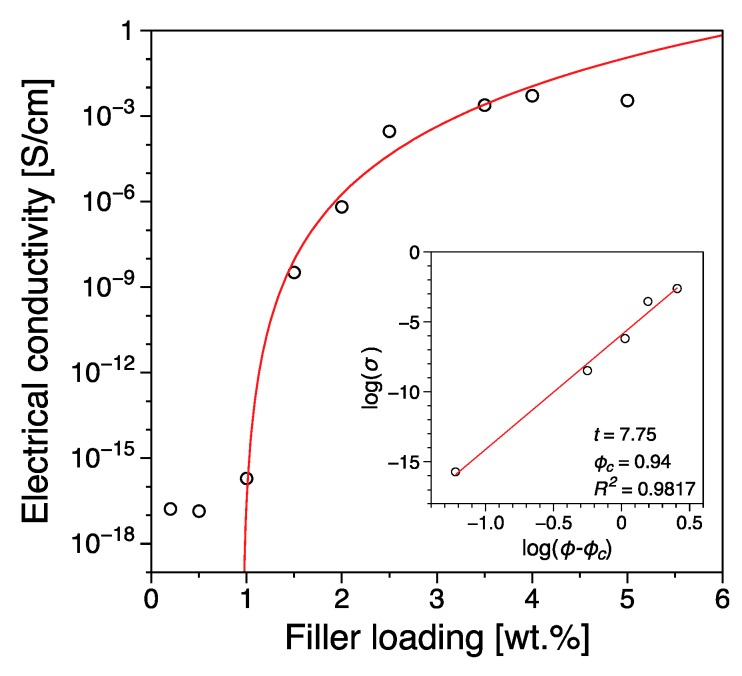
Electrical conductivity as a function of filler loading for the iPP-based nanocomposites. The insert shows the power-law application to the experimental data for evaluation of the electrical percolation threshold and the associated parameters obtained. An average error of ~4% is inferred for the reported values of electrical conductivity.

In Figure 3 the electrical conductivity as a function of the filler loading can be seen. For low amounts of graphene (0.1–0.8 wt.%) we observe conductivities very close to those of the pure polymer of the order of 10^−16^ S/cm. This indicates that although a network is in the process of being formed, it is far from complete, thus restricting the charge movement through the material. Beyond ~1 wt.% we observe a sharp increase in the observed conductivity value with some early indications of a plateau for higher concentrations of the order of 3–5 wt.%. The system’s conductivity follows a percolation-type behavior [52]

$$\sigma =\sigma_{o}{\left(\phi -\phi_{C}\right)}^{t}$$

with *σ_o_* a pre-exponential factor that is dependent on the conductivity of the filler, the network topology and the types of contact resistance. The terms ϕ and $\phi_{C}$ correspond to the filler concentration and the critical concentration at the transition (also known as percolation threshold) and *t* the critical exponent. The percolation threshold was estimated at 0.94 wt.% with a critical exponent of *t* = 7.75. We have measured each sample three times in order to establish the reproducibility of our results and we have observed an average error of ~4% for the reported values of electrical conductivity. The value of the critical exponent observed in this study is significantly higher from the theoretical value ~2 for three dimensions [46,53]. According to percolation theory for a single percolation the critical exponent is dependent only on the dimensionality of the system [53], although non-universal behavior has been reported for experimental nanocomposites with values varying from 1 to 11 [54]. The universality of the critical exponent has been confirmed by a number of numerical calculations of random resistor models [54]. The reason for the significant disparity of the critical exponent value reported in this study with respect to the theoretical predictions is difficult to pinpoint. However, it could be associated with the existence of a multi-step percolation behavior where a tunneling mechanism dominates the current flow between the conductive particles [46,55].

Most importantly, the percolation threshold reported in this study is significantly lower than those reported previously for polypropylene [40,41,56] using melt mixing methods [27] and other similar polymers [57]. An overview of the reported electrical percolation values for a number of different polypropylene/graphene systems prepared with various melt mixing protocols is presented in Table 1. The significantly lower value of the percolation threshold reported in this study indicates a superior level of dispersion, something especially interesting if we take into consideration that no pre- or post-mixing procedures were used. Furthermore, the entire mixing and preparation protocol was intentionally kept as simple as possible aiming to resemble a potential commercial mixing process.

### 3.2. Morphology

**Figure 4:**
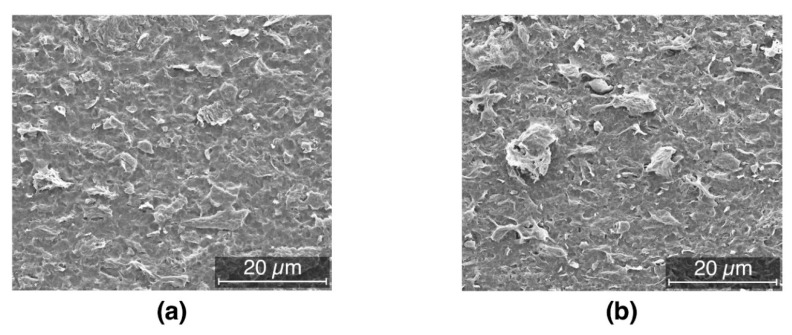
SEM images after etching, showing (**a**) isolate rare agglomerates (1 wt.% filler) or (**b**) larger structures (2.5 wt.% filler).

Despite the superior dispersion and the very low filler content needed for electrical percolation, a level of agglomeration and aggregation of the filler is still present as indicated by the SEM images of different compositions reported in Figure 4. The composition with 1 wt.% content exhibits limited agglomeration. This further indicates the substantial potential of the filler to achieve electrical percolation at even lower content once dispersion is further optimized. It is worth remembering that in this work the mixing process was kept as simple as possible, i.e., eliminating all the pre- and post- mixing stages in order to simulate a composite production process that is as similar as possible to large-scale industrial procedures. Still despite these very severe restrictions, the level of dispersion can be considered efficient as seen from the low value of the electrical percolation threshold.

**Figure 5:**
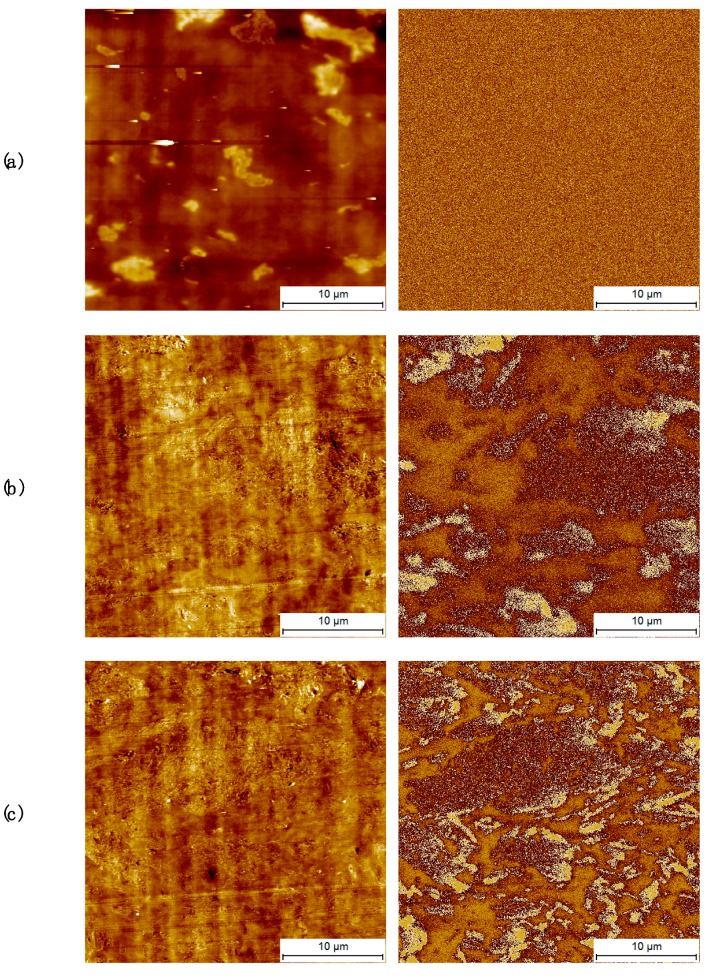
Electrostatic force microscope (EFM) images of iPP with (**a**) 1 wt.%, (**b**) 2.5 wt.%, (**c**) 5 wt.% filler. The left column shows standard AFM topography images, while the right column shows EFM nap-phase images.

In order to further understand the effect of agglomerates on the electrical conductivity and the extent of the percolation network, images were collected by electrostatic force microscope (EFM). In the vicinity of the macroscopic electrical percolation, the phase images of the EFM mode exhibit only noise, indicating the lack of a well-formed network and of electrical interaction with the tip voltage, despite the clear presence of agglomerates visible in the topography image, Figure 5a. As the network expands and the observed value of the electrical conductivity increases, significant contrast is observed in the EFM phase images, Figure 5b,c. In particular, two different regions can be observed in these images, namely the matrix and the filler agglomerates. Enrichment of charges in the bright area cause the electrical interaction with the AFM tip, in particular disturbing it therefore creating a phase contrast, thus indicating that different parts of the filler contribute differently to the conduction process.

**Figure 6:**
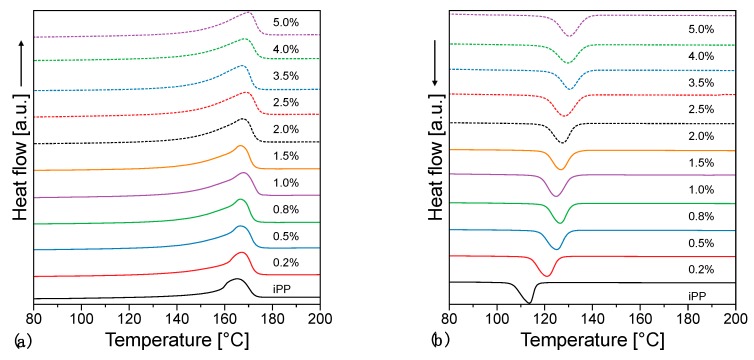
DSC (**a**) second heating and (**b**) cooling thermograms.

Polypropylene has a very rich polymorphism form and it has been reported that filler inclusion has an effect of the overall morphology of the system [56]. For this reason, we have investigated the effect of the filler in the overall crystallinity and the resulting morphology. Crystallisation and second heating thermograms can be seen in Figure 6 and the overall values for crystallinity are summarized in Table S1. From this, we can clearly see that the crystallization temperature increases with increasing filler loading. At low loadings especially, the increase is quite intense as with just 0.2 wt.% of filler the crystallization temperature increases by 7 °C in comparison with the value observed for the pure polymer (113.6 °C to 120.8 °C). This behavior can be associated with nucleation effects and has been reported in the past for a number of fillers including clay, nanotubes and graphitic platelets [35,61,62].

**Figure 7:**
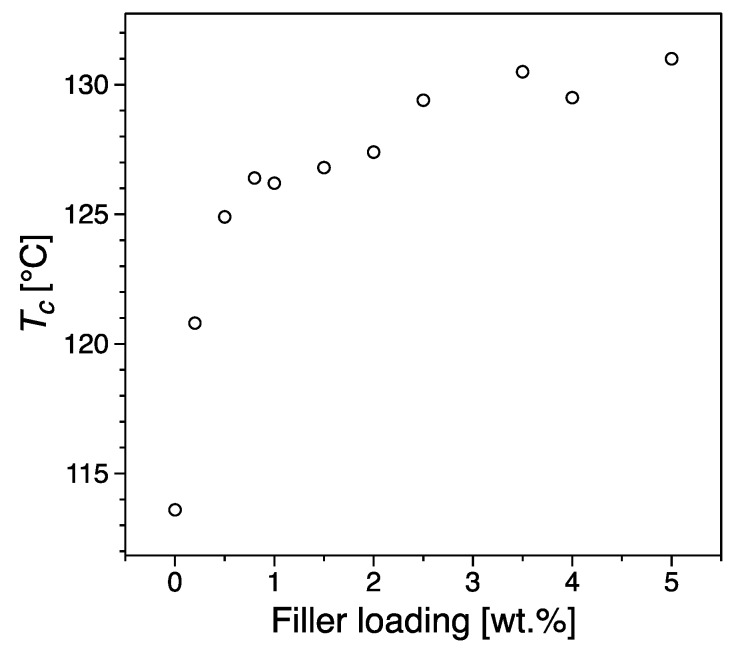
Crystallization temperature, *T_c_*, as a function of the filler loading.

**Figure 8:**
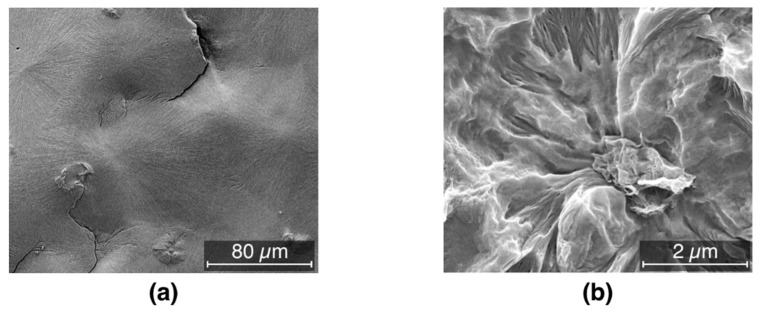
SEM images of (**a**) large spherulites obtained from the pristine material and (**b**) lamellae growing from such an inclusion.

The crystallization temperature, taken as a proxy for nucleation efficiency, increases with increasing filler loading. In particular the plot of Figure 7 is typical of the addition of nucleating agents, with a steep increase of T_c_ which saturates when the nucleating effect is complete. Figure 8a shows the morphology obtained for the pristine material, characterized by large spherulites due to the low nucleation rate of such a pure material. On the other hand, Figure 8b shows an example of crystalline lamellae growing from a graphene inclusion. Remarkably, the crystallization temperature of a fully nucleated sample is ca. 130 °C, as typical of the best nucleating agents commercially available [63]. This behavior has been reported in the past for a number of fillers including clay, nanotubes and graphitic platelets [35,55,64], and most recently by Beuguel et al. [65].

**Figure 9:**
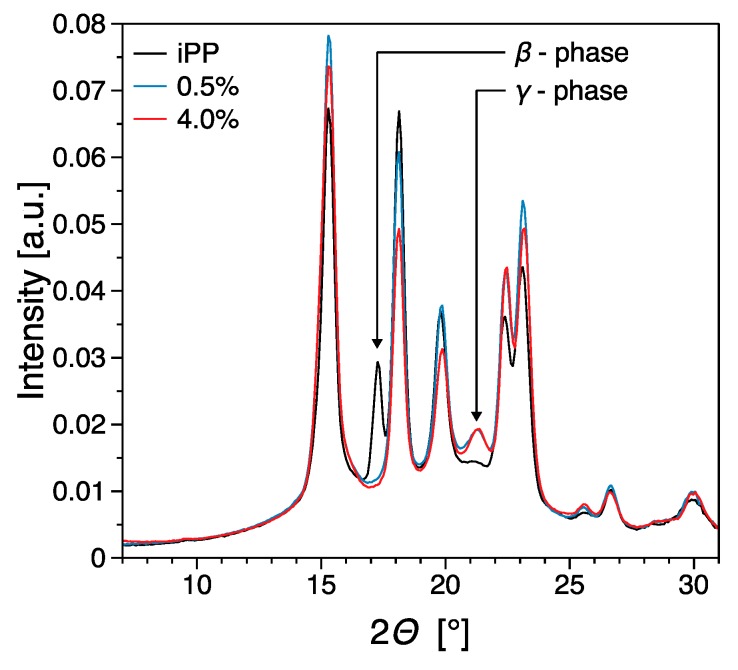
WAXS spectra of the nanocomposites investigated at selected representative concentrations.

Polypropylene has a very rich polymorphism and it has been reported that filler inclusion has an effect of the overall morphology of filled systems [39]. The iPP used in this work shows generally a large tendency to form crystals in β phase, mostly due to the high purity of this system [66]. The presence of β phase for the pure material is clearly observed from the [300]_β_ peak in Figure 9. Upon nucleation with the filler however β phase disappears, unlike the work of Zhao [67]. Instead, relatively large amounts of *γ* phase are obtained as visible from the [117]*_γ_* peak in Figure 9. Most of these findings are in agreement with the results by Beuguel et al. [65], who showed epitaxial growth of crystals from graphene nanoplatelets. This is also shown in the SEM micrograph of Figure 8b, and in particular transcristallinity from the oriented agglomerates, however no *γ* phase was observed in that work. The reasons for this difference can be tentatively attributed to differences in the type of filler used and possibly in the iPP used, since nucleation of *γ* phase is not surprising for metallocene PP, due to the presence of the effects of regiodefects and for PP copolymers with ethylene, however nucleation of γ phase for this iPP in particular in this work, is not something that is commonly observed [63,68].

### 3.3. Thermal Properties

**Figure 10:**
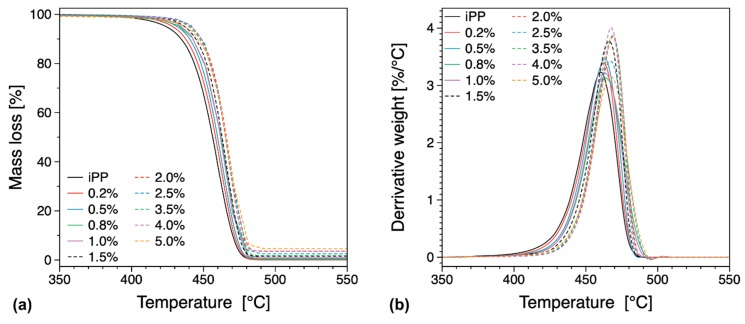
TGA (**a**) and differential thermo-gravimetric (DTG) (**b**) curves for the iPP composites with various filler loadings (in wt.%).

Thermal stability and the amount of filler in the different compositions used in this study were evaluated from TGA and differential thermo-gravimetric (DTG) curves (see Figure 10 and Table S2). From these results, we can see that the temperature corresponding to half the decomposed sample *T_max_* is increasing with increasing filler loading, indicating an improvement in the thermal stability of the composition. This improvement in thermal stability can be associated with the inherent property of graphene to act as a mass transport barrier that inhibits the transport of polymer degradation by-products to the surface [43]. Traditionally, the polymer decomposition at the initial stages has been reported to reduce the material thermal stability when carbonaceous fillers are used [43,56,69]. In this work such behavior is not observed as we can see the general trend of increasing thermal stability to be maintained even at low loadings (see Figure S1). For a pure polymer thermal decomposition will be initiated at the surface of the sample, whereas if thermally conductive filler particles are present the heat and the associated thermal decomposition will be transferred into the bulk in a more efficient manner, thus leading to a situation where thermal decomposition occurs both at the surface and the bulk of the sample under investigation. Graphene has been reported of having superior barrier properties [2,3], therefore it is expected that it can improve the material thermal stability by creating a tortuous path to the various volatiles that are created by thermal decomposition in the bulk of the sample. In other words, we can expect two competing mechanisms to be present namely efficient heat transfer into the bulk of the sample and creation of barriers that can slow down thermal decomposition [56]. In our system, we observe that even at low filler loadings of the order of 0.2 or 0.5 wt.% the thermal stability of the material is substantially increased indicating that the material benefits from the existence of a tortuous path created by the filler. Since the loadings are very low and below the electrical percolation threshold (see previous discussion on the electrical properties) the extent of the network created by the filler is fairly limited. Therefore, we can assume that the level of dispersion is significant and thus capable to create an extended network-like structure that can limit the volatile dispersion and increase the thermal stability.

### 3.4. Thermal Conductivity

**Figure 11:**
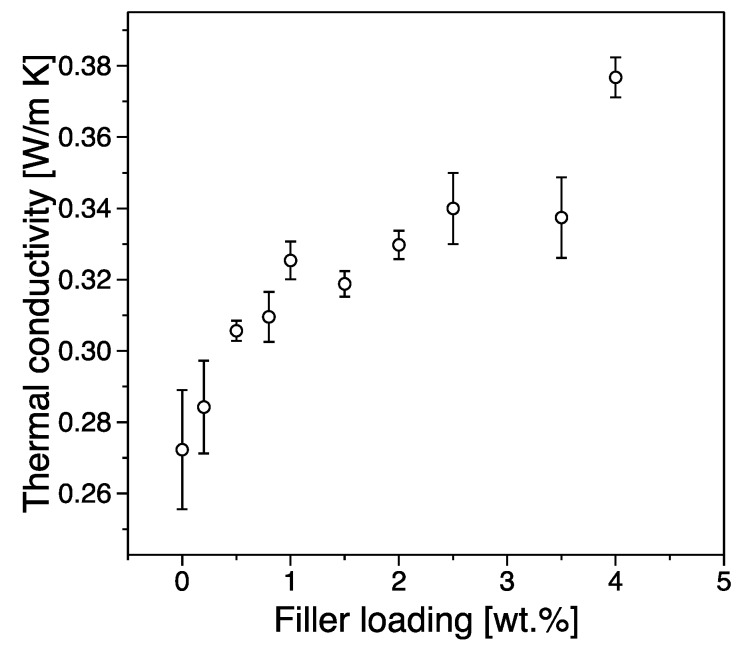
Thermal conductivity vs filler loading.

Figure 11 shows the thermal conductivity as a function of filler content. The incorporation of graphene significantly enhances the thermal conductivity of iPP, and the thermal conductivity increases steadily with increasing filler content. From our results we can see that the observed thermal conductivity increases from 0.27 Wm^−1^K^−1^ for pure polypropylene to 0.37 Wm^−1^K^−1^ for a system with 4 wt.% filler exhibiting an increase of ~37%. Heat propagation in graphene/iPP nanocomposites is generated mainly due to acoustic phonons and electrons scattering processes. The effective heat conduction of the composite depends on the thermal resistance between filler and the polymer matrix and scattering processes on its interface as well as potential thermally conductive paths created by the existence of the filler network. We can observe a rapid increase in thermal conductivity with increasing filler loading below the electrical percolation threshold indicating that although the overall network is still being created, the dispersion process is efficient enough to allow for such processes to occur. Above the electrical percolation threshold, the increase in thermal conductivity is more gradual, something that can be associated with the possibility of agglomeration and aggregation processes that increase with the extension of the network formation in the matrix. In composite materials, the scattering of phonons is due to the existence of a thermal barrier, originating from an acoustic mismatch at the interface between the organic polymer and the inorganic filler. Therefore, high filler loadings compared to the electrically conductivity threshold (i.e., >25 wt.%) are typically necessary to achieve an appropriately high level of thermal conductivity. Such an increased loading has the potential to represent a significant processing challenge and can in certain conditions cause mechanical properties of interest to deteriorate [42].

### 3.5. Mechanical Properties

#### 3.5.1. Melt Rheology

**Figure 12:**
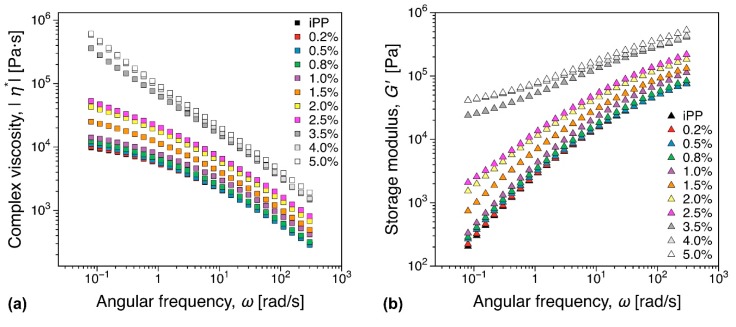
Dynamic frequency sweep test showing (**a**) the complex viscosity and (**b**) storage modulus increase with increasing filler content.

**Figure 13:**
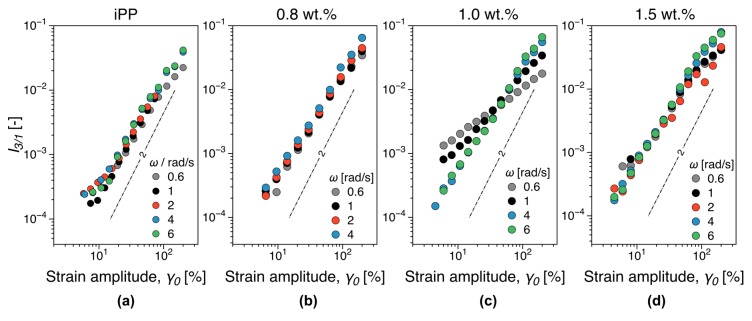
Third relative harmonic, *I_3/1_*, in strain sweep tests (**a**) for the iPP matrix, (**b**) below percolation (0.8 wt.%), (**c**) at the electrical percolation (1 wt.%), and (**d**) above percolation (1.5 wt.%). The instrument noise at low strain amplitudes has been excluded from the graphs, see Figure 2.

The addition of fillers can significantly increase the melt viscosity and affect polymer chain mobility [70]. Therefore, rheological properties are important for industrial applications primarily as means to assess the nanocomposite processability. Here we focus on two aspects: (i) The increase in complex viscosity and (ii) percolation threshold. Linear viscoelastic dynamic frequency sweep measurements for all concentrations investigated are shown in Figure 12. It should be noted that the empirical Cox–Merz rule is generally not valid for filled polymer systems [71]. However, the complex viscosity at a constant angular frequency ω= 60 rad/s can be considered as a measure of the increase in viscosity, as it is at sufficiently high ω to approach the shear thinning region, while sufficiently low that similar data would be available in most comparable studies. A plot of the relative increase in complex viscosity can be found in the Supplementary information—see Figure S2. Close to the electrical percolation threshold, a 1.42 fold increase in complex viscosity is observed, value comparable to viscosity increase reported for MWCNT nanocomposites [72]. In the conductor region a factor of 7.5 increase in complex viscosity was recorded for the highest iPP filler concentration (5%). In contrast, carbon black alternatives can reach 40% in filler concentration and increase the complex viscosity with a factor of up to 10 [72], significantly hindering their processability. The storage modulus is a typical linear viscoelastic indicator for the existence of a percolated filler network within the polymer as the additional elastic contribution of the filler network can be revealed through the existence of a plateau (solid-like behavior) in the limit of vanishing angular frequencies. In the angular frequencies range investigated, a concentration of over 2.5 wt.% could be there as identified as a threshold concentration. This is significantly higher than the electrical percolation threshold of the iPP nanocomposites, see Figure 3. The third relative higher harmonic, *I_3/1_*, is shown in Figure 13 at selected representative concentrations. For concentrations below 1 wt.%, the nonlinear scaling is similar to the general behavior outlined in Section 2.6, see Figure 2b, for the iPP (compare also Figure 13a,b). In contrast, a significant qualitative change in the nonlinear material response is recorded at 1 wt.%. Thus, the *I_3/1_* scaling in the MAOS region becomes angular frequency dependent with angular frequencies greater than 2 rad/s reverting to $I_{3/1}\propto \gamma_{0}^{2}$ as it is the case for the iPP. This potentially corresponds to the existence of a weakly formed filler network that is disrupted by the shear flow. A more thorough analysis and further discussion on the interpretation of the nonlinear data in terms of rheological and electrical percolation thresholds, as well as potential nanocomposite morphological fingerprinting is discussed elsewhere [73].

#### 3.5.2. DMTA

**Figure 14:**
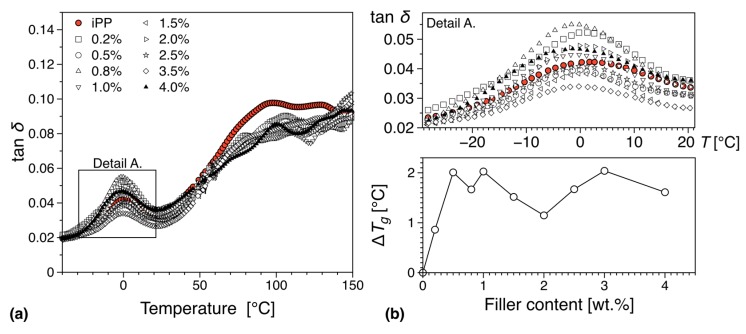
Glass transition temperature, *T_g_*, variation from dynamic mechanical thermal analysis (DMTA) tests: (**a**) tan(δ) as function of temperature and (**b**) variation in *T_g_* as function of filler content.

In Figure 14 we can see the mechanical loss tangent (tan(*δ*)) behavior as a function of temperature and filler loading. For the case of pure polypropylene, three molecular relaxations have been reported in the literature, and these transitions namely α, β, and γ are observed with decreasing temperature [74]. For completion, we must note that a fourth relaxation (δ-relaxation) has been reported from dielectric spectroscopy data at −173.15 °C [75,76]. In this work, we have performed DMTA in the region −40 °C to 200 °C, and therefore the γ and δ relaxations are not visible.

**Figure 15:**
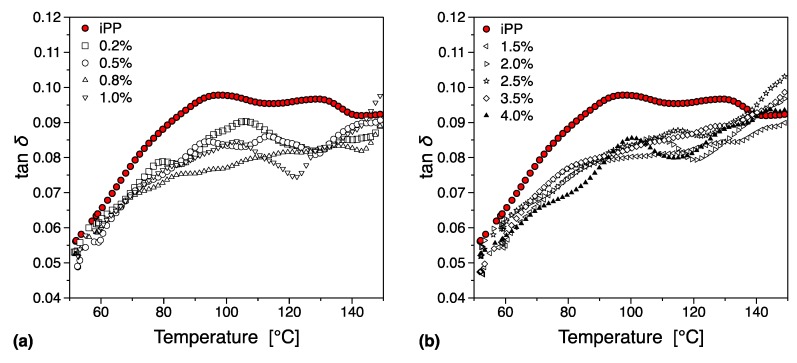
α relaxation below (**a**) and above (**b**) percolation threshold.

The β-transition is thought to being related to segmental relaxation mechanisms in the amorphous region, and has been associated with the glass transition [77], something that has been confirmed by its higher activation energy in comparison with the α-relaxation [78]. From this work we can see that the inclusion of the filler decreases the observed glass transition (see Figure 14 and Figure 15), something that can be associated with the existence of interfacial interactions between the filler surface and the polymer matrix. These can take the form of Hydrogen bonds and can increase the relaxation mechanism, leading to an overall decrease in *T_g_*. Furthermore, it is worth noticing that there appears to be a gradual decrease in the observed glass transition temperature below the electrical percolation threshold. This rate of change remains relatively unchanged in the region above the electrical percolation, indicating that despite the increased amount of available filler no further interaction can be observed, probably due to agglomeration or localized saturation of the available graphene surfaces. Previous work in different PMMA isomers with different graphene types and preparation conditions has indicated a correlation between the level of dispersion and the strength of the interaction between the filler and the matrix to the observed glass transition temperature [79].

In comparison to polyethylene [80,81,82], the mechanical α-relaxation of polypropylene is less investigated. The common view is that the origin of this transition can be associated to relaxations on both the amorphous and the crystalline phases of the matrix, with the overall relaxation divided into two separate ones α_1_ and α_2_ that are located at lower and higher temperatures respectively [83]. The exact origin of the different relaxations is still under investigation, but it is believed that both α_1_ and α_2_ can be associated with specific intralamellar motions and other intracrystalline processes, originating from the existence of anisotropies on the crystal lattice potential [84]. The α relaxation can be in general associated with the exchange of isotactic segments between amorphous and crystal phases, with chain conformation, molecular architecture, molecular weight and processing conditions known to have a significant influence [85].

From this work we observe a significant suppression of the overall α-process with increasing filler loading, something that can be associated with the morphological changes the introduction of the filler initiates in the matrix (See Figure 14 and Figure 15). As we have seen in our earlier discussion the introduction of the filler leads to significant morphological changes in the matrix due to the high extent of nucleation. These changes and the overall increase of the γ-phase can be associated with the changes in the α transition we observe. Furthermore, it is interesting to note that the suppression of the α relaxation is more pronounced for compositions above the electrical percolation threshold (see Figure 15) where the individual transitions α_1_ and α_2_ almost disappear. This behavior can be associated with the overall morphological changes discussed previously, namely the suppression of the β phase and the increased nucleation activity [86]. Since below the electrical percolation these changes are more gradual and the overall level of agglomeration less prominent one can expect that a more subtle change in the overall behavior of the α relaxation. Above the electrical percolation we have a fully formed network that has provided all the nucleation activity that is capable of and with each increase in filler loading we observe a slight but important increase in the overall agglomeration level.

## 4. Conclusions

In this work, we have presented an industrially relevant melt-mixed system of polypropylene and hierarchical reduced graphene oxide. The filler allows for the creation of a three-dimensional extended structure, something that leads to efficient dispersion. This efficiency can be seen via the low electrical percolation threshold observed by this study. Furthermore, it must be noted that we have kept the entire mixing procedure as simple as possible, something that was seen to have adverse effects on the overall filler dispersion in the past [1,2,3]. In this study despite the simple mixing method we observe a percolation threshold several times lower than those reported in the literature, indicating the existence of a superior dispersion process. This allows us to gain partial access to the individual filler particles, something that was so far possible only for solution and other advanced mixing methods, thus obtaining some of the unique properties of high aspect ratio fillers for a melt mixed system. We believe that the combination of these purpose-built graphenes with industrially relevant polyolefins have the potential to pave the road for future solutions that will be able to deliver breakthrough performance while surpassing the trade-offs that plague systems based on conventional fillers.

## Figures and Tables

**Table 1:** Comparison of previously reported results on electrical percolation threshold of polypropylene prepared by various melt mixing methods.

Filler Type	Percolation Threshold [wt.%]	Reference
Reduced Graphene Oxide	0.94	this work
Graphite Platelets	9	[47]
Expanded Graphite	8–12	[58]
Graphene nanoplatelets	8–6	[40]
Graphene nanoplatelets	8	[59]
Graphite nanoplatelets	12	[42]
Graphene nanoplatelets	8–12	[60]

## Supplementary Materials

The following are available online at https://www.mdpi.com/2079-4991/9/12/1766/s1, Figure S1: The initial stages of the thermal decomposition of the various concentrations, Figure S2: Relative increase in complex viscosity with increasing filler content. The data corresponds to a constant angular frequency of 60 rad/s in Figure 13a. Table S1: DSC Data summary of the melting and crystallization temperatures identified. Table S2. Thermal stability temperature as a function of filler loading.
