# Supplementary material for: Melt-Mixed 3D Hierarchical Graphene/Polypropylene Nanocomposites with Low Electrical Percolation Threshold
Source: Nanomaterials (Basel). 2019 Dec 11;9(12):1766. doi: 10.3390/nano9121766 (PMC6956219; doi:10.3390/nano9121766)
Supplement: Supplementary file 1 [file nanomaterials-09-01766-s001.pdf]

nanomaterials

Article

Melt-mixed 3D Hierarchical Graphene/Polypropylene Nanocomposites with Low Electrical Percolation Threshold

Thomas Gkourmpis [1,*], Karolina Gaska [2,†], Davide Tranchida [3], Antonis Gitsas [3], Christian Müller [4], Aleksandar Matic [5] and Roland Kádár [2]

1 Innovation & Technology, Borealis AB, SE-444 86 Stenungsund, Sweden
2 Department of Industrial and Materials Science, Engineering Materials, Chalmers University of Technology, SE-412 96 Gothenburg, Sweden; karolina.gaska@bristol.ac.uk or gaskak@chalmers.se (K.G.); roland.kadar@chalmers.se (R.K.);
3 Innovation & Technology, Borealis Polyolefine GmbH, St.-Peter-Straße 25, 4021 Linz, Austria; davide.tranchida@borealisgroup.com (D.T.); antonis.gitsas@borealisgroup.com (A.G.)
4 Department of Chemistry and Chemical Engineering, Chalmers University of Technology, SE-412 96 Gothenburg, Sweden; christian.muller@chalmers.se
5 Department of Physics, Chalmers University of Technology, SE-412 96 Gothenburg, Sweden; matic@chalmers.se
* Correspondence: thomas.gkourmpis@borealisgroup.com; Tel.: +46-303-205-576
† Present Address: Department of Aerospace Engineering, University of Bristol, Bristol BS8 1 TR, UK.

Received: date; Accepted: date; Published: date

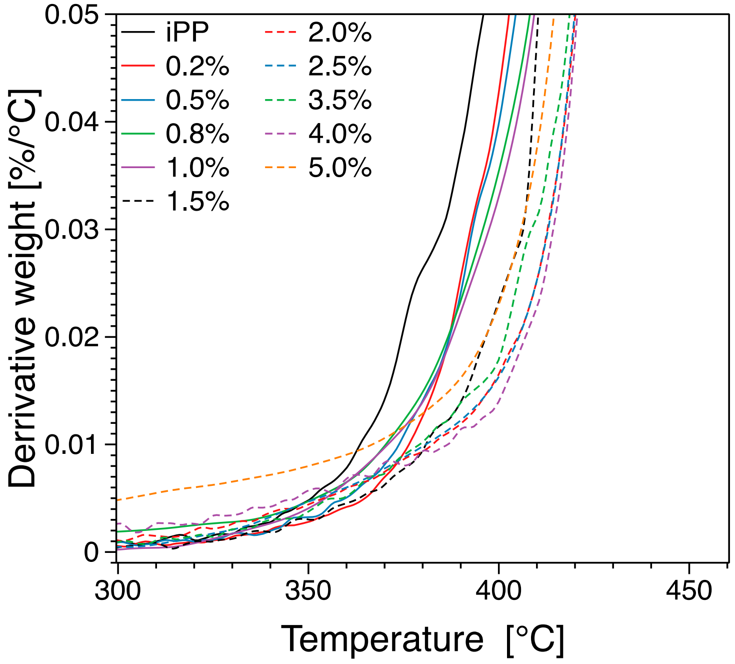

Figure S1. The initial stages of the thermal decomposition of the various concentrations.

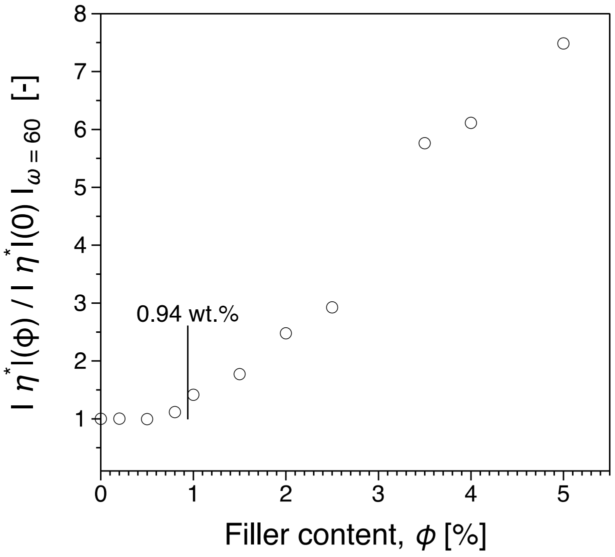

Figure S2. Relative increase in complex viscosity with increasing filler content. The data corresponds to a constant angular frequency of 60 rad/s in Figure 13a.

Table S1. DSC Data summary of the melting and crystallisation temperatures identified.

Filler Loading [wt.%]	T_m [°C]	T_C [°C]	χ [%]	ΔH [J/g]
0	165.2	113.6	53.0	109.6
0.2	167.2	120.8	55.1	114.3
0.5	166.5	124.9	55.3	115.0
0.8	166.6	126.4	55.5	115.8
1	167.2	126.2	52.6	110.1
1.5	166.6	126.8	53.6	112.6
2	167.5	127.4	53.2	112.4
2.5	168.0	129.4	53.1	112.8
3.5	167.4	130.5	48.4	103.9
4	168.4	129.5	50.3	108.4
5	169.2	131.0	50.8	110.8

Table S2. Thermal stability temperature as a function of filler loading.

Filler Loading [wt. %]	T_{max} [°C]
0	459.6
0.2	462.1
0.5	463.2
0.8	463.6
1	463.6
1.5	466.1
2	468.7
2.5	467.1
3.5	468.8
4	467.7
5	467.9

© 2019 by the authors. Submitted for possible open access publication under the terms and conditions of the Creative Commons Attribution (CC BY) license (http://creativecommons.org/licenses/by/4.0/).
